# A Systematic Review and Meta‐Analysis of the Efficacy of Antimicrobial Chemoprophylaxis for Recurrent Acute Otitis Media in Children

**DOI:** 10.1111/coa.14240

**Published:** 2024-10-12

**Authors:** Timothy Davies, Xicheng Peng, Joseph Salem, Zeynep C. Elcioglu, Anna Kremneva, Mei‐yin Gruber, Kristijonas Milinis, Michael W. Mather, Jason Powell, Sunil Sharma

**Affiliations:** ^1^ Department of Paediatric Otolaryngology Alder Hey Children's Hospital Liverpool UK; ^2^ Translational and Clinical Research Institute Newcastle University Newcastle upon Tyne UK; ^3^ Department of Otolaryngology St Georges University Hospital NHS Foundation Trust London UK; ^4^ Department of Paediatric Otolaryngology Great North Children's Hospital Newcastle upon Tyne UK; ^5^ Biosciences Institute Newcastle University Newcastle upon Tyne UK

**Keywords:** acute otitis media, antimicrobial, chemoprophylaxis, children, middle ear

## Abstract

**Objectives:**

Acute otitis media (AOM) is a common childhood infection. Recurrent AOM affects a subset of children, resulting in an adverse impact on quality of life, socioeconomic disadvantage, and risk of long‐term sequelae. Antimicrobial chemoprophylaxis is used in some settings but is increasingly controversial due to an awareness of adverse long‐term effects and contribution to global antibiotic resistance.

**Design and Setting:**

A comprehensive literature search was undertaken using Medline (1946–October 2023) and Embase (1974–October 2023). The primary aim was to assess the efficacy of antimicrobial chemoprophylaxis on AOM episodes in children < 18 years of age. Bias and quality assessment was performed. Dichotomous data were analysed using risk ratio with 95% confidence intervals. Meta‐analysis was carried out using random‐effects models for pooled analysis, independent of heterogeneity. Heterogeneity was assessed using the *I* ^2^ statistic.

**Main Outcome Measures:**

The effect of antimicrobial chemoprophylaxis in children with rAOM on the number of individual AOM episodes. Secondary outcomes: assessment of antimicrobial agents and outcomes in children with risk factors.

**Results:**

Assessment of qualitative data was performed on 20 studies (*n* = 2210). No controlled trials were identified post‐multivalent pneumococcal conjugate vaccine (PCV) introduction, restricting current generalisability. Quantitative meta‐analysis on nine pre‐PCV studies (*n* = 1087) demonstrated antimicrobial chemoprophylaxis reduced any episode of AOM with a risk ratio 0.59 (95% CI 0.45–0.77).

**Conclusion:**

Families and clinicians must balance marginal short‐medium term benefit (based on pre‐PCV data), and the potential for adverse effects to that individual, and the societal risk of antimicrobial resistance with prolonged antibiotic use.

**Summary**

- Acute otitis media (AOM) is one of the most common infections of childhood. Recurrent AOM affects a subset of children, causing substantial socioeconomic implications.
- Multivalent pneumococcal conjugate vaccine (PCV) introduction has resulted in alteration in the incidence and predominant otopathogens of AOM.
- Our current evidence base for the use of antimicrobial chemoprophylaxis in recurrent AOM is based on limited pre‐PCV data from over two decades ago.
- Understanding of the adverse effects associated with antibiotic over use and the contribution to global antibiotic resistance has increased in recent years.
- Laboratory and clinical studies are required to better understand AOM aetiology and the interplay with other middle ear conditions, such as otitis media with effusion and chronic otitis media.

## 1 Introduction

Acute otitis media (AOM) is defined as the presence of inflammation in the middle ear, associated with rapid onset of symptoms and signs of an ear infection [1]. AOM is one of the most common infections of childhood and is a leading cause for antibiotic prescribing in economically developed countries [1, 2]. AOM can result in long‐term sequelae, such as hearing loss and tympanic membrane perforation [3]. Life‐threatening complications, such as mastoiditis and brain abscesses, while rare, can result in significant morbidity and mortality, particularly in low‐income countries [3, 4, 5].

Recurrent AOM (rAOM) has multiple definitions, however, the most commonly used is; three or more well‐documented and separate AOM episodes in the preceding 6 months or four or more episodes in the preceding 12 months, with at least one episode in the past 6 months [6]. At 2 years of age, up to 5% of all children will have experienced rAOM [7], impacting on the quality of life for children and their carers [6, 8, 9, 10]. There are substantial socioeconomic implications of these recurrent infections, with frequent medical visits, prescriptions and indirect costs relating to lost days of work for carers [8, 11, 12]. In the United States (US), from 2014 to 2018, rAOM in children cost an estimated $1.3 billion per year [13].

Antimicrobial chemoprophylaxis is one of the possible interventions for rAOM, along with ventilation tube insertion and observation [1]. *Streptococcus pneumoniae*, *Haemophilus influenzae* and *Moraxella catarrhalis* are the three most common otopathogens associated uncomplicated AOM [14]. The microbiology of AOM with tympanic membrane perforation includes a greater proportion of S. *pyogenes* and *Staphylococcus aureus* [15, 16, 17]. From 2009 onwards, multivalent pneumococcal conjugate vaccines (PCVs) have been licenced and implemented in many nationwide immunisation programmes worldwide [18]. This has resulted in alteration in the incidence and frequency of AOM and changes in the predominant otopathogen (towards *H*. *influenzae* and non‐PCV serotypes amongst other changes) [12, 19, 20, 21, 22, 23, 24, 25]. Recent estimates suggest AOM disease burden remains high in children of all ages despite overall reductions in AOM incident rates during PCV introductions [26].

There is little description of current practice, or guidelines, in the searchable literature related to antimicrobial chemoprophylaxis in rAOM [6, 27, 28, 29]. A recent United Kingdom survey of practice suggested antimicrobial chemoprophylaxis is offered by up to 70% by otolaryngologists [29]. Whereas US 2013 and Italian 2019 paediatric guidelines advice against its routine use [6, 28] Spanish 2023 guidelines suggest antimicrobial chemoprophylaxis as an option, alongside ventilation tubes and observation [27]. Further to this, when chemoprophylaxis is used the antimicrobial agent, duration of prophylaxis and drug dosing regimen is highly variable in the published literature [1, 29, 30]. Antimicrobial chemoprophylaxis is increasingly controversial due to the adverse lifelong effects associated with antibiotic use in early childhood and the potential contribution to global antibiotic resistance [31, 32, 33, 34, 35].

The aim of this systematic review was to summarise and characterise the existing literature addressing the use of antimicrobial chemoprophylaxis in paediatric rAOM.

## 2 Materials and Methods

### 2.1 Protocol and Registration

The protocol was published on PROSPERO (CRD42024487812) and was reported according to the Preferred Reporting Items for Systematic Reviews and Meta‐Analysis (PRISMA) guidelines [36].

### 2.2 Study Search Strategy

A comprehensive search was undertaken using Medline (1946–October 2023) and Embase (1974–October 2023) up to and including October 2023. Keyword search included the search term ‘otitis media’ AND (‘anti‐infective agents’, ‘antibiotic prophylaxis’, ‘anti‐bacterial agents’ and ‘anti‐bacterial agents’) AND ‘recurrence’.

### 2.3 Inclusion and Exclusion Criteria

Search results were limited to English language and human only studies. Narrative reviews, editorials, case reports, duplicate publications, qualitative studies, conference abstracts and non‐human studies were excluded. Studies were limited to paediatric populations (< 18 years of age) and those published in English language. The titles and abstracts were screened and full‐text copies of all the articles deemed potentially relevant were retrieved. A broad definition of rAOM was used in the initial search, with no criterion placed on frequency of rAOM episodes.

### 2.4 Outcomes

The primary objective was to assess the effect of antimicrobial chemoprophylaxis in children with rAOM on the number of individual AOM episodes. Secondary outcomes included, sub‐group analysis of only studies meeting the commonly used American Academy of Otolaryngology‐Head and neck Surgery (AAO‐HNS) definition of rAOM at preventing any further episodes of AOM [6]. Additionally, sub‐group analysis of differing antimicrobial agents used and outcomes in children with associated risk factors.

### 2.5 Risk of Bias and Quality of Evidence Assessment

The risk of bias was assessed by two independent reviewers (XP and MM) using the Cochrane ‘risk of bias tool for randomised trials’ (RoB2) and ‘risk of bias in non‐randomised studies of interventions’ (ROBINS‐I) tools for randomised and non‐randomised studies, respectively [37, 38]. A third reviewer (JP) additionally evaluated any cases where discrepancy occurred between these assessments. Plots were created using robvis, which is a statistical web application for visualising risk‐of‐bias assessment [36]. An overall assessment of the evidence quality for outcome measures was reported according to the Grading of Recommendations, Assessment, Development and Evaluations (GRADE) assessment [39]. The software program GRADEpro was used [40].

### 2.6 Statistical Analysis

The meta‐analysis was conducted using Review Manager, OpenMeta (Analyst) and R Meta package [41, 42, 43]. A *p* value of less than 0.05 was accepted to be statistically significant. Dichotomous data were analysed using risk ratio (RR) with 95% confidence intervals (CIs). Random‐effects models for pooled analysis was used, independent of heterogeneity. Heterogeneity was assessed using the *I* ^2^ statistic.

## 3 Results

### 3.1 Study Selection, Study Characteristics, Participant Characteristics, Study Interventions and Controls

**FIGURE 1:**
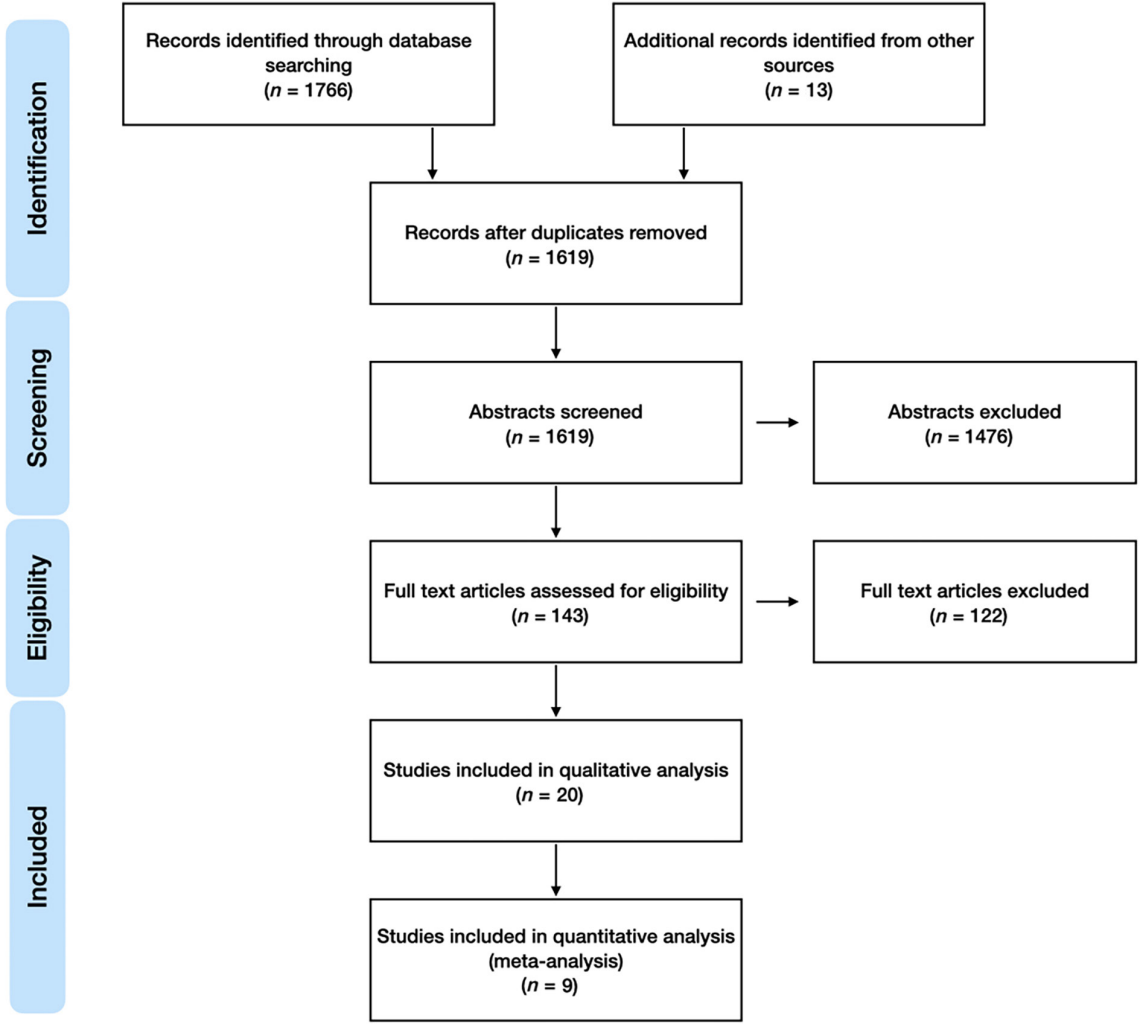
Flow diagram of systematic review identification, screening, eligibility assessment and inclusion.

The literature search findings are summarised in Figure 1. In total 20 studies (*n* = 2210 children, median 81 participants per study [IQR 54–157, range 21–364]) were included in qualitative analysis, of which nine were suitable for quantitative meta‐analysis (Table 1) [22, 44, 45, 46, 47, 48, 49, 50, 51, 52, 53, 54, 55, 56, 57, 58, 59, 60, 61, 62, 63]. Only two non‐controlled studies reported participants recruited in the post‐PCV era [47, 63]. The majority of studies used the Lieberthal et al. [6] definition of rAOM as criteria for inclusion, defined as three or more well‐documented and separate AOM episodes in the preceding 6 months or four or more episodes in the preceding 12 months with at least one episode in the past 6 months [22, 44, 46, 47, 50, 51, 52, 53, 57, 58, 62, 63]. The most common intervention in the treatment group was the use of amoxicillin chemoprophylaxis (10/20 studies) [22, 44, 46, 47, 53, 57, 58, 60, 61, 63]. The most frequent control intervention was placebo (12/20 studies) [22, 49, 50, 52, 54, 55, 57, 58, 59, 60, 61, 62], followed by tympanostomy tube insertion (2/20 studies) [22, 49]. The follow‐up period and duration of therapy was variable, median 6 (IQR 4–11.6, range 1–48) months, median 4 (IQR 3–6, range 1–48) months, respectively. Compliance with treatment, often by measurement of medication volume, a medication diary or urine analysis, was quantified in 10 studies (50%) [22, 50, 53, 54, 55, 57, 58, 59, 61, 62], of which the rates were generally high. Complications of the antimicrobial treatment was described in five studies (25%), of which the rates were low [22, 48, 50, 59, 61].

**TABLE 1:** Overall study characteristics.

Reference	Study design	Blinding	Randomisation	Country of origin	Year of study commencement	Year of study conclusion	Lower age limit (months)	Upper age limit (months)
Vijayakumar and Santhi 2019	Comparative study	N	Y	India	2013	2015	24	156
Ellul et al. 2018	Case series	N	N	UK	2010	2012	ND	ND
Koivunen et al. 2004	Controlled trial	Double	Y	Finland	1994	1999	10	24
De Diego et al. 2001	Comparative study	ND	Y	Spain	1998	1999	9	120
Teele et al. 2000	Controlled trial	Double	Y	USA	ND	ND	ND	ND
Roark and Berman 1997	Controlled trial	Double	Y	USA	ND	ND	3	72
Marchisio et al. 1996	Comparative study	Single	Y	Italy	1992	1996	9	60
Sih et al. 1993	Controlled trial	ND	Y	Brazil	ND	ND	9	108
Casselbrant et al. 1992	Controlled trial	Double	Y	USA	1981	1988	7	35
Berman 1992	Comparative study	Single	Y	USA	1998	1990	ND	ND
Principi et al. 1989	Controlled trial	Single	Y	Italy	1986	1988	9	60
Gonzalez, Arnold, Erhardt 1986	Controlled trial	Double	Y	USA	1982	1985	6	120
Lampe and Weir 1986	Crossover trial	ND	ND	USA	1981	1983	0	72
Varsano, Volovitz, and Mimouni 1985	Crossover trial	Double	Y	Israel	ND	ND	6	60
Liston and Harbison 1984	Retrospective comparative study	ND	N	USA	ND	ND	0	60
Schwartz, Puglise, Rodriguez 1982	Crossover trial	Double	Y	USA	1979	1980	4	72
Gaskins et al. 1982	Controlled trial	ND	Y	USA	ND	ND	12	168
Biedel 1978	Controlled trial	ND	N	USA	ND	ND	ND	ND
Perrin et al. 1974	Crossover trial	Double	Y	USA	1972	1973	11	96
Maynard, Fleshman, and Tschopp 1972	Controlled trial	Double	N	USA	ND	ND	0	84

Reference	No. of participants	Intervention groups	Comparator groups	Complications reported	Outcome measure	Follow up period (months)	Prophylaxis period (months)
Vijayakumar and Santhi 2019	86	Amoxicillin or azithromycin	No therapies	ND	Recurrence following prophylactic treatment with azithromycin and amoxicillin in recurrent AOM Efficacy of amoxicillin and azithromycin between age groups of children	6	6
Ellul et al. 2018	41	Amoxicillin	No therapies	ND	Comparison of children responding to those not responding to the 6 week course of antibiotics Effect of exposure to environmental cigarette smoke	6.2	1.4
Koivunen et al. 2004	180	Sulfafurazole	Adenoidectomy, placebo	Y (9%)	Intervention failure (2 episodes in 2 months or 3 episodes in 6 months or persistent effusion) during follow up	24	6
De Diego et al. 2001	69	Azithromycin or amoxicillin	No therapies	ND	Effectiveness of the prophylaxis categorised by 50% or more reduction in number of AOM episodes Comparison of effectiveness between azithromycin and amoxicillin	11.5	3
Teele et al. 2000	117	Amoxicillin or sulfisoxazole	Placebo	Y (ND)	Experience with acute otitis media post entry into study	12	4.8
Roark and Berman 1997	158	Amoxicillin	Placebo	ND	Effectiveness of continuous once vs. twice daily dosing in preventing new episodes of otitis media	1	ND
Marchisio et al. 1996	157	Amoxicillin or azithromycin	No therapies	ND	Occurrence of AOM compared between amoxicillin and azithromycin during the prophylaxis period Occurrence of AOM during prophylaxis in relation to epidemiological factors Occurrence of AOM compared between amoxicillin and azithromycin during the post‐prophylaxis period	12	6
Sih et al. 1993	60	Amoxicillin or sulfamethoxazole/trimethoprim	Placebo	ND	Number of AOM recurrences Number of recurrences by month	3	3
Casselbrant et al. 1992	264	Amoxicillin	Tympanostomy Tube Placebo	Y (7%)	Rates of new episodes of either acute otitis media or otorrhea after entry Rates of new episodes of otitis media after entry	48	48
Berman 1992	75	Amoxicillin	No therapies	ND	Effectiveness of continuous versus intermittent amoxicillin to prevent episodes of otitis media	4	4
Principi et al. 1989	96	Amoxicillin or sulfamethoxazole/trimethoprim	Placebo	N	Occurrence of acute otitis media during study period Occurrence of acute otitis media in relation to epidemiological factors	6	6
Gonzalez, Arnold, Erhardt 1986	63	Sulfisoxazole	Tympanostomy Tube Placebo	ND	Results by treatment for prevention of recurrent acute otitis media	6	6
Lampe and Weir 1986	87	Erythromycin or sulfisoxazole	No therapies	ND	Outcome by 2 month periods of observation with or without episodes of otitis media while on erythromycin versus no prophylaxis Outcome of patients and attack rate in episodes per 2‐months period prior to, during and following prophylaxis with erythromycin and sulfisoxazole	8	4
Varsano, Volovitz, and Mimouni 1985	32	Sulfisoxazole	Placebo	N	Occurrence of acute otitis media with sulfisoxazole and placebo therapy Occurrence of acute otitis media with sulfisoxazole and placebo therapy related to sequence of treatment regimens	4.6	2.3
Liston and Harbison 1984	52	Sulfisoxazole	Placebo	ND	Effectiveness of chemoprophylactic administration of sulfisoxazole Epidemiologic factors and rates of otitis media	3	3
Schwartz, Puglise, Rodriguez 1982	33	Sulphamethoxazole	Placebo	Y (3%)	Number of episodes of otitis media during treatment with sulphamethoxazole compared with placebo	4	2
Gaskins et al. 1982	21	Trimethoprim/sulfamethoxazole	No therapies	Y (30%)	Difference in number of recurrences before and after entry into the study	6	6
Biedel 1978	201	Sulfisoxazole or trisulfapyrimidines	Decongestants	ND	Number of recurrent otitis cases following an URI	1.8	1.8
Perrin et al. 1974	54	Sulfisoxazole	Placebo	ND	Episodes of otitis media during sulfisoxazole versus placebo therapy Attack rate of otitis media according to age during the study	6	3
Maynard, Fleshman, and Tschopp 1972	364	Ampicillin	Placebo	ND	Number of otitis media during study year according to type of medication	12	12

### 3.2 Episodes of Acute Otitis Media With Antimicrobial Chemoprophylaxis

**FIGURE 2:**
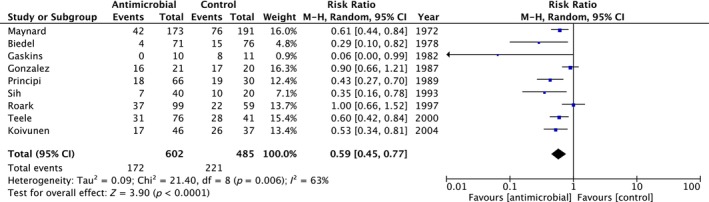
Forest plot and accompanying data from studies included within the meta‐analysis demonstrating a reduced risk of recurrent episodes of AOM in children treated with prophylactic antibiotics compared with placebo or control. Including all study defined definitions of recurrent AOM.

The study primary outcome measure was number of AOM episodes in all studies and nine studies were included in the primary meta‐analysis [45, 48, 49, 50, 54, 57, 58, 60, 61]. All studies without a control comparator group (*n* = 6), crossover studies (*n* = 4) and studies that did not quote absolute numbers for use in the quantitative analysis (*n* = 1) were excluded. Initial analysis included any study defined definition of rAOM. Long term antibiotics reduced any episode of AOM with a risk ratio (RR) of 0.59 (95% CI 0.45–0.77, *I* ^2^ = 63%, *p* < 0.05, *n* = 1087 children) (Figure 2). Using cumulative event rates (control [221 out of 485 participants] vs. antibiotic [172 out of 602 participants]), the absolute risk reduction (ARR) is 17% and the number needed to treat (NNT) is 5.88. This means that approximately six children would be needed to be treated long‐term to prevent one child experiencing AOM while on treatment. Given the significant study‐related heterogeneity across all included studies, further sub‐group analysis was carried out.

### 3.3 Prevention of Any Further Episodes of Acute Otitis Media With Antimicrobial Chemoprophylaxis (Using Established Literature Definition of rAOM)

**FIGURE 3:**
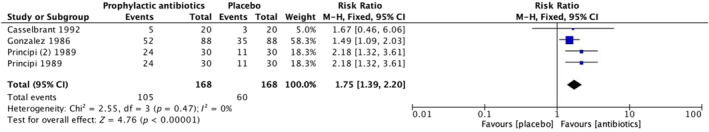
Forrest plot and accompanying data from studies included within the meta‐analysis demonstrating the efficacy of prophylactic antibiotics compared with placebo or control in preventing all further episodes of recurrent AOM. Including only studies meeting American Academy of Paediatric definition of recurrent AOM.

Sub‐group meta‐analysis of studies using the established literature definition of rAOM was undertaken. Three studies with four treatment arms met this criteria [15, 22, 49]. One study, previously excluded in the initial meta‐analysis, was reintroduced, as it met inclusion criteria for sub‐group analysis [22]. The mean duration of treatment in these included studies was 18.6 weeks (IQR 15–24, range 4–26). The mean duration of follow up was 52 weeks (IQR 26–65, range 26–104). Analysis was undertaken to establish the impact of chemoprophylaxis on the proportion of patients with no further episodes of rAOM following completion of treatment. The pooled RR was 1.75 (CI 1.39–2.20, *I* ^2^ = 0, *p* < 0.001 *n* = 315 children), favouring prophylactic antibiotic group (Figure 3).

### 3.4 Comparison of Effectiveness Between Different Chemoprophylaxis Agents

**FIGURE 4:**
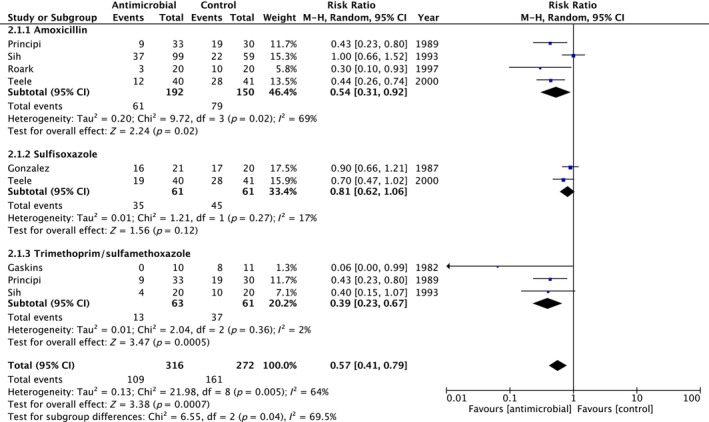
Forest plot and accompanying data from studies demonstrating individual and overall effect of selected antibiotic treatments on reducing risk of recurrent acute otitis media versus control.

A number of trials reported the comparison between different agents used (*n* = 7) [46, 51, 53, 57, 60, 61, 63]. Sub‐group meta‐analysis (included any study defined definition of rAOM) of antimicrobial agents demonstrated that trimethoprim and sulfamethoxazole was the most likely intervention to reduce AOM episodes RR 0.39 (95% CI 0.23–0.67, *I* ^2^ 2% *p* = 0.36, *n* = 124, three studies) compared with amoxicillin RR 0.54 (95% CI 0.31–0.92, *I* ^2^ 69% *p* = 0.02, *n* = 242, four studies) and sulfamethoxazole RR 0.81 (95% CI 0.62–1.06, *I* ^2^ 17% *p* = 0.27, *n* = 122, two studies) (Figure 4). Comparing treatment with trimethoprim and sulfamethoxazole versus sulfamethoxazole alone demonstrate RR 0.58 (95% CI 0.36–0.95, *I* ^2^ = 69%, *p* = 0.01, five studies), with a sub‐group difference of *χ* ^2^ = 5.77 (*p* = 0.02, *I* ^2^ = 82.7%).

### 3.5 Recurrent Acute Otitis Media and Associated Risk Factors

**FIGURE 5:**
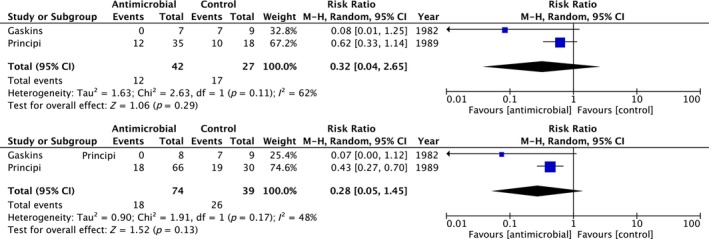
Forest plot and accompanying data from studies demonstrating individual and overall effect of antibiotic treatments on reducing incidence of recurrent acute otitis media versus control in specifically (A) males and (B) children aged 5 years and under.

AOM episodes were reported in relation to possible risk factors such as age [22, 44, 47, 48, 52, 53, 55, 57, 62, 63], cigarette smoke exposure at home [47], sex [22, 47, 48, 52, 53, 57, 63], season [22, 53, 57, 58], attendance at day‐care [47, 52, 53, 57] and history of atopy [52]. Sub‐group meta‐analysis (included any study defined definition of rAOM) was limited by study data, but was performed in groups with known risk factors including males; RR 0.32 (95% CI 0.04–2.65, *I* ^2^ 62% *p* = 0.11, *n* = 69, two studies), and children 5 years or younger; RR 0.28 (95% CI 0.05–1.45, *I* ^2^ 48% *p* = 0.17, *n* = 113, two studies) (Figure 5).

## 4 Discussion

In conclusion, we present an updated review of the literature related to antimicrobial chemoprophylaxis in rAOM. Although broadly evidence from the pre‐PCV era (and two small non‐randomised studies from the post‐PCV era) indicate that antimicrobial chemoprophylaxis is associated with a modest reduced frequency of AOM whilst taking prophylaxis, and a greater proportion of patients with no further episodes of rAOM following completion of treatment. There are, however, several overriding limitations to the available data, which restrict the generalisability of these findings to current practice.

Firstly, it is important to acknowledge that most of the available data is based on populations studied prior to introduction of the PCV, which may have impacted the incidence and microbiology of AOM [19, 20, 21, 22, 23, 64]. Two uncontrolled studies recruiting in the post PCV era, a 2019 study [63] of Azithromycin once weekly versus amoxicillin daily and a 2015 case series of amoxicillin prophylaxis [47] are difficult to interpret in isolation. The vast majority of our evidence on this topic is based on decades old studies with little additional since the 2006 Cochrane review on by Leach and Morris, which summarised acute and chronic suppurative otitis media studies [30]. Furthermore, the studies definition of rAOM, and interventions, such as antimicrobial agent, control, dose range and duration of prophylaxis were highly heterogeneous. The populations studied also varied substantially, for example by age and geographical location. The latter is important as there is likely to be variation in pathogenic organisms related to AOM between regions and associated antibiotic susceptibilities [14, 65]. Risk of bias was present amongst all included studies (Figures S1 and S2). Areas of high risk of bias or uncertainty often related to missing data or bias in measuring/reported outcomes. The restrictive design of some of the studies also limited the generalisability of the findings. Funnel plots were visually inspected for identification of publication bias and demonstrated a high risk of publication bias (Figure S3). The GRADE quality of evidence was moderate for our primary meta‐analysis outcome measure of AOM episodes and low to very low for secondary outcomes (Figure S4).

All study primary outcome measures were related to episodes of AOM. Both our outcomes of reduced AOM episodes on treatment and absence of episodes post‐treatment demonstrated a modest improvement with chemoprophylaxis. While these are important outcome measures, more subtle patient reported outcome measures that capture the severity of individual episodes or quality of life implications would additionally inform more about the effect these interventions have on children and carers [5, 10, 66]. For example, missed school days for children and time of work for carers [10]. Furthermore, economic evaluation of the impact of these interventions would be beneficial in terms of the cost of this intervention versus alternatives, such as conservative management or ventilation tubes, both at a healthcare and societal level [13, 67]. In the UK, the National Institute for Health and Care Excellence (NICE) does not recommend antimicrobial prophylaxis in primary care in most circumstances therefore secondary care appointments are recommended prior to starting this intervention [68]. rAOM can overlap with other middle ear pathology such as otitis media with effusion (OME) [69], but we found no data on the impact of antibiotics on other important outcomes such as speech, language and cognitive development. Finally, microbiological outcomes, including resistance patterns, were infrequently reported in the described studies, which would be an important potential further outcome measure with prolonged antimicrobial use.

Antimicrobial chemoprophylaxis for rAOM must be considered in the context of alternatives treatments. While alternative interventions exist, namely, ventilation tubes, their effectiveness is also likely marginal and they pose risks such as chronic perforation of the tympanic membrane. A Cochrane systematic review from 2018 on this topic summarised five RCTs with unclear or high risk of bias [70]. All were conducted prior to the introduction of pneumococcal vaccination. Based on what they deemed to be very low‐quality evidence, they concluded that it was uncertain whether ventilation tubes were more effective than antibiotic prophylaxis at preventing rAOM. Ventilation tubes were however superior to active monitoring and placebo medication. A 2021 randomised controlled trial by Hoberman et al. published after this review randomly assigned 250 children (6–35 months of age) with rAOM to either undergo ventilation tube placement or receive medical management, involving episodic antimicrobial treatment [71]. The rate of AOM was not significantly lower with ventilation tube placement than with medical management. However, the placement of ventilation tube does offer opportunity for treatment of AOM with topical therapy through the tube, which may reduce the selection pressure on antibiotic resistance compared with oral administration [72]. The other option is conservative management with episodic management of AOM [1]. Antimicrobials are frequently used as part of AOM management, despite the majority of episodes of AOM being non‐bacterial and their use having limited effect on symptoms [65, 73]. The NNT to prevent suppurative complications is large, but concern related to this and wanting to alleviate parental concern is high [73, 74, 75]. A Cochrane review summarised low quality evidence indicating that both paracetamol and ibuprofen are effective in relieving short‐term ear pain in children with AOM [76]. They also highlight the need for research into other analgesics, such as anaesthetic eardrops [76, 77, 78].

There are several modifiable risk factors in rAOM management, including avoiding exposure to passive smoking and ensuring children have a complete course of vaccinations [79]. Screening tests for an adequate antibody response to Haemophilus influenzae type B (Hib) and pneumococcal vaccines, are available in many high resource settings now, as well as screening for immune deficiencies, such as immunoglobulin blood levels, or more extensive immune investigations [20, 80]. Only one study included within this meta‐analysis specifically addressed a high‐risk population group, however, several studies did attempt a sub‐group analysis of populations with known risk factors in their analysis. Children without associated co‐morbidities can still have risk factors, including young age and male sex [79, 81, 82]. Additionally, polymorphisms in genes involved in immune pathways that predispose to rAOM [83, 84].

Even in situations where is a clearly demonstrated benefit of antimicrobials, intervention must always be carefully balanced against the risk of adverse effects and the emergence of antimicrobial resistance [32, 33]. The risk of antimicrobial resistance has been specifically linked to the widespread use of antibiotics for common conditions such as AOM [65, 85, 86]. RAOM poses a challenge when considering antimicrobial stewardship, as many children receive multiple courses of treatment dose antibiotics for individual episodes of AOM [29, 74, 75]. Therefore, prophylactic treatment must be considered in the context of its ability to reduce the episodes of treatment dose interventions. Furthermore, the impact of antimicrobial chemoprophylaxis must also be considered at both an individual level and at a societal level [87]. The effect of antimicrobials on the lifetime risk of multiple conditions, from atopy, autoimmune disease and cancer to depression and obesity, are not fully elicited, and likely dependant on multiple factors, including the age of the child and duration of antimicrobial treatment [31, 88].

New PVCs containing a greater number of pneumococcal serotypes are in stages of development [89, 90]. This will likely lead to further evolution of the AOM phenotype, requiring additional evaluation of the effectiveness of various interventions [91]. Laboratory and clinical trials are required to understand better how continuous low‐dose antibiotic prophylaxis works and to find ways of maximising benefit while minimising antimicrobial resistance and associated harm. This balance is likely to involve well‐designed trials, careful study of population groups, as well as translational study of the interplay between bacteria and host responses [14, 35, 69, 92, 93].

## 5 Conclusions

An awareness of antimicrobial stewardship amongst clinicians and parents is become more widely accepted [31, 32, 33, 34, 35]. Currently, a balance must be made between short‐medium term benefit of antimicrobial chemoprophylaxis in reduced AOM episodes, and the potential for long‐term individual and societal harm. Improved risk stratification of the children most likely to benefit from interventions will hopefully evolve out of better designed clinical, laboratory and epidemiological studies. Interventions targeting modifiable risk factors and new novel therapies will optimistically become increasingly at the forefront of early management.

## Author Contributions

All authors meet the definition of authorship established by ICMJE. All authors involved in conceptualization, literature review, manuscript formulation and manuscript revision.

## Ethics Statement

The authors have nothing to report.

## Conflicts of Interest

The authors declare no conflicts of interest.

## Supporting information


**Figure S1.** Risk of bias in randomised studies included within the meta‐analysis. Assessed using the revised Cochrane risk of bias tool for randomised trials (RoB2) tool [37].

**Figure S2.** Risk of bias in non‐randomised studies included within the meta‐analysis. Assessed using the Risk Of Bias In Non‐randomised Studies—of Interventions (ROBINS‐I) tool [38].

**Figure S3.** Funnel plot demonstrating high level of bias amongst studies providing data included within the meta‐analysis.

**Figure S4.** Summary of *Grading of Recommendations*, *Assessment*, *Development and Evaluations* (GRADE) assessment of outcomes [39, 40]. CI: confidence interval; RR: risk ratio; (a) see separate bias assessments (Figures S1 and S2), (b) wide confidence intervals represents imprecision of estimate of effect, (c) discordant results between studies.

## Data Availability

The data that support the findings of this study are available from the corresponding author upon reasonable request.
