# Supplementary figures and images for: A Systematic Review and Meta‐Analysis of the Efficacy of Antimicrobial Chemoprophylaxis for Recurrent Acute Otitis Media in Children
Source: Clin Otolaryngol. 2024 Oct 12;50(1):1–14. doi: 10.1111/coa.14240 (PMC11618227; doi:10.1111/coa.14240)

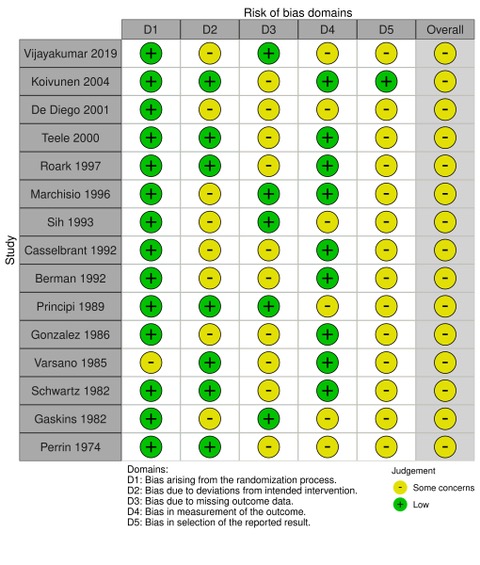

Supplement: Supplementary file 1 — Figure S1. Risk of bias in randomised studies included within the meta‐analysis. Assessed using the revised Cochrane risk of bias tool for randomised trials (RoB2) tool [37]. [file COA-50-1-s001.jpeg]

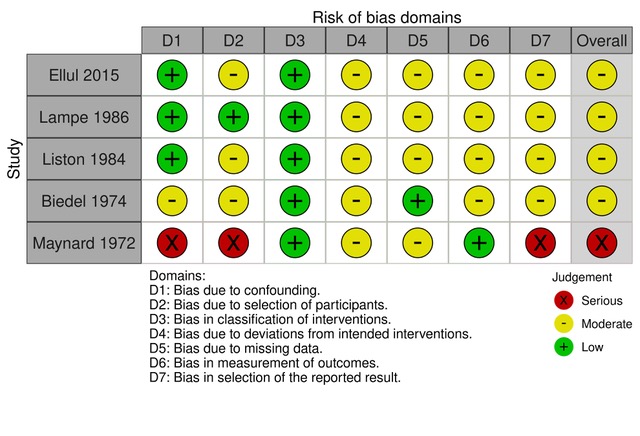

Supplement: Supplementary file 2 — Figure S2. Risk of bias in non‐randomised studies included within the meta‐analysis. Assessed using the Risk Of Bias In Non‐randomised Studies—of Interventions (ROBINS‐I) tool [38]. [file COA-50-1-s004.jpeg]

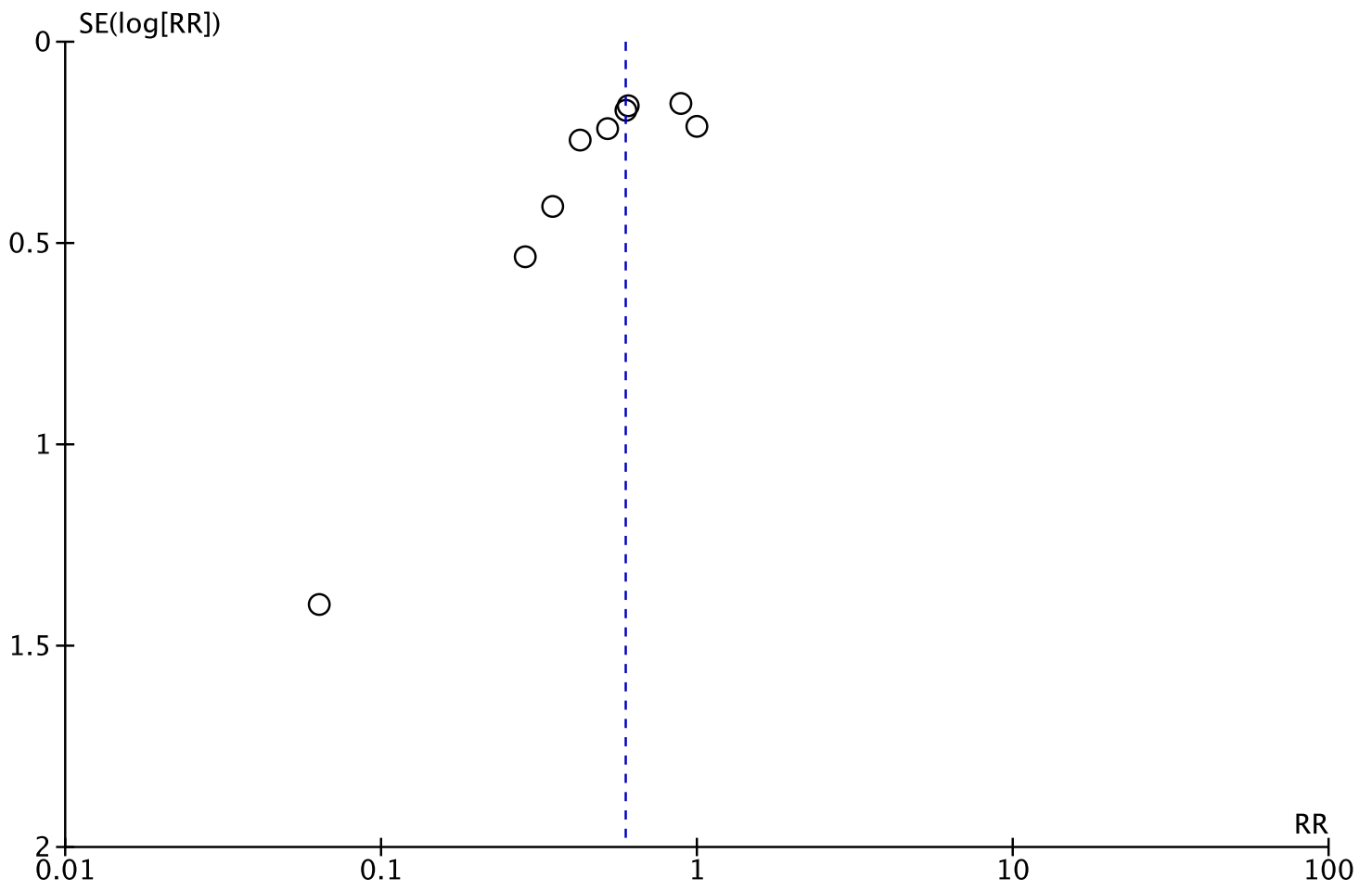

Supplement: Supplementary file 3 — Figure S3. Funnel plot demonstrating high level of bias amongst studies providing data included within the meta‐analysis. [file COA-50-1-s002.pdf]

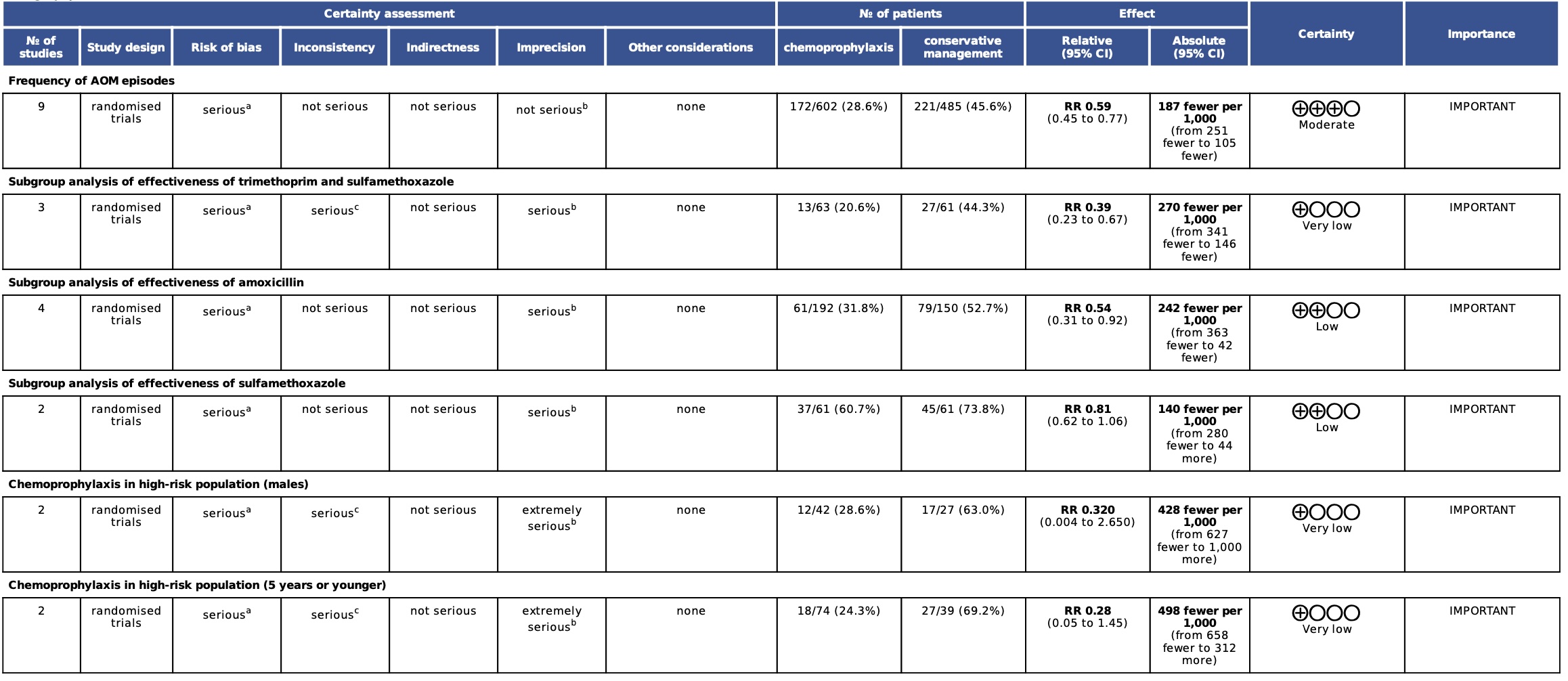

Supplement: Supplementary file 4 — Figure S4. Summary of Grading of Recommendations, Assessment, Development and Evaluations (GRADE) assessment of outcomes [39, 40]. CI: confidence interval; RR: risk ratio; (a) see separate bias assessments (Figures S1 and S2), (b) wide confidence intervals represents imprecision of estimate of effect, (c) discordant results between studies. [file COA-50-1-s003.jpg]
